# An Electrostatic Method for Manufacturing Liquid Marbles and Particle-Stabilized Aggregates

**DOI:** 10.3389/fchem.2018.00280

**Published:** 2018-07-10

**Authors:** Peter M. Ireland, Casey A. Thomas, Benjamin T. Lobel, Grant B. Webber, Syuji Fujii, Erica J. Wanless

**Affiliations:** ^1^Priority Research Centre for Advanced Particle Processing and Transport, University of Newcastle, Callaghan, NSW, Australia; ^2^Department of Applied Chemistry, Faculty of Engineering, Osaka Institute of Technology, Osaka, Japan; ^3^Nanomaterials Microdevices Research Center, Osaka Institute of Technology, Osaka, Japan

**Keywords:** liquid marble, electrostatics, air-water interface, adsorption, particle

## Abstract

We have developed a method for transferring particles from a powder bed to a liquid droplet using an electric field. This process has been used to create liquid marbles with characteristics not normally found in those formed by direct contact methods such as rolling. It has also been used to manufacture hydrophilic particle-liquid aggregates and more complex layered aggregates incorporating both hydrophobic and hydrophilic particles. This article briefly outlines the electrostatic aggregation method itself, the materials used and structures formed thus far, and explores the rich fundamental physics and chemistry underpinning the process as they are understood at present.

## Introduction

**Figure 1:**
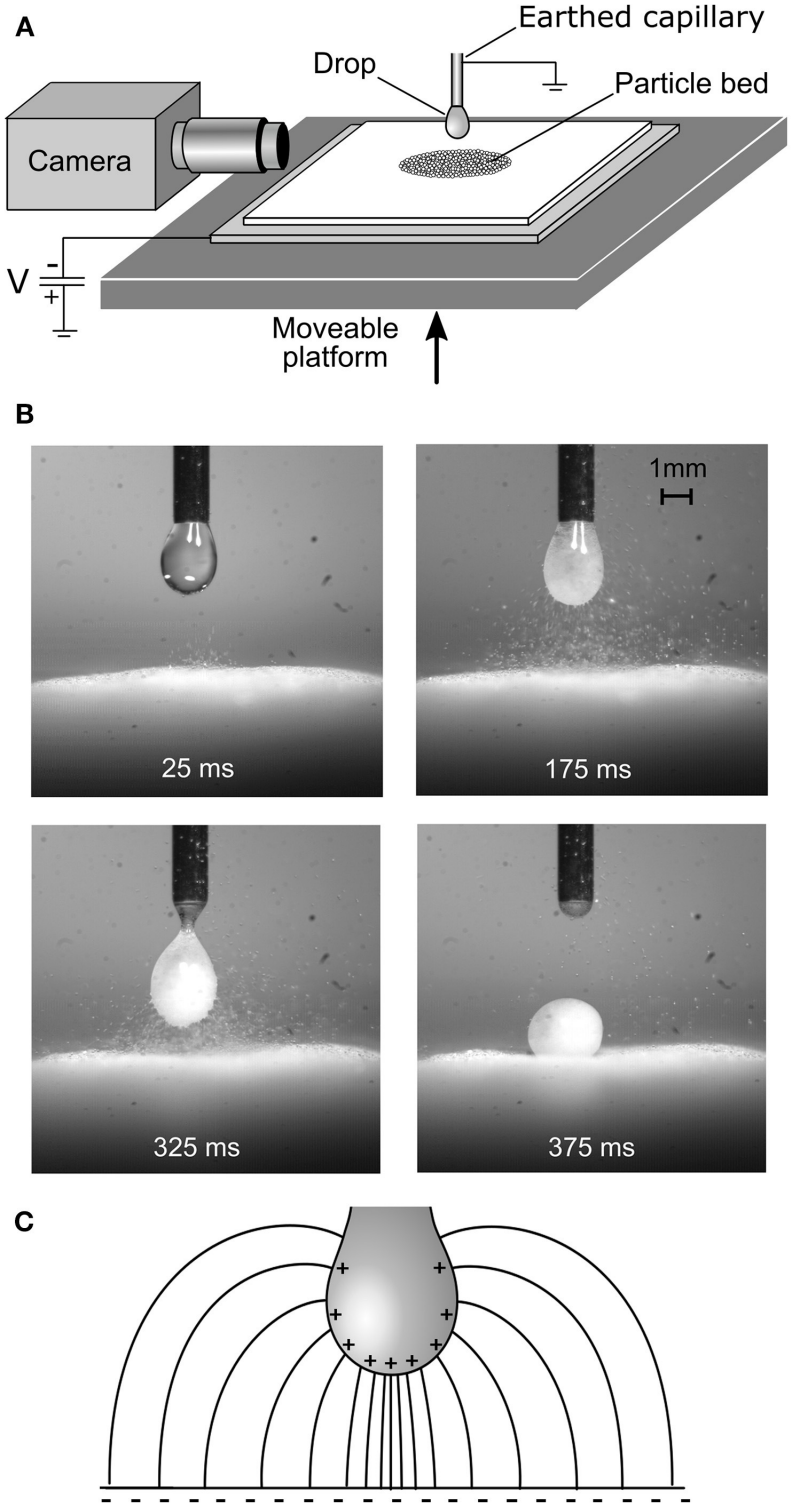
**(A)** Schematic of the electrostatic aggregation method. **(B)** Formation of a metastable aggregate with hydrophilic ballotini. **(C)** Schematic of the converging shape of the electric field lines between the substrate and drop.

Drop-particle aggregates were first manufactured by our group using an electrostatic process in 2013 (Liyanaarachchi et al., 2013). In that early study, a bed of 100 μm diameter ballotini (glass spheres) was placed on a polytetrafluoroethylene (PTFE) coated glass slide, which had been negatively tribocharged (charged by rubbing). When the particle bed was brought near a water droplet suspended from a capillary, the glass spheres suddenly jumped to the drop in a vigorous “avalanche.” Being hydrophilic, the particles were internalized by the drop, filling it like a “sack of marbles,” and forming a pearl-like liquid-particle aggregate (Figure 1) on top of the particle bed. In subsequent studies, the tribocharged PTFE was replaced by a metal plate connected to a high voltage DC power supply, allowing a fully controllable driving potential.

A superficial resemblance was quickly noted between the liquid-particle aggregates formed in this early study, and the liquid marbles (LMs) first described nearly two decades ago (Aussillous and Quéré, 2001). The main difference was that our electrostatically-formed aggregate was a drop entirely filled with hydrophilic particles, whereas liquid marbles consist of a drop encased in hydrophobic particles. The resemblance was nonetheless of interest, since LMs display some remarkable properties, e.g., extreme recoverable deformability, low evaporation rate, and the ability to come into non-wetting contact with solid surfaces and to float on water (McHale and Newton, 2011, 2015; Janardan et al., 2015). These properties have in turn inspired a range of proposed applications for LMs, including gas sensors (Tian et al., 2010), bioreactors (Arbatan et al., 2012a,b), encapsulation media (Eshtiaghi et al., 2010; Ueno et al., 2014), pressure-sensitive adhesives (Fujii et al., 2016a) and materials delivery carriers (Paven et al., 2016; Kawashima et al., 2017).

**Figure 2:**
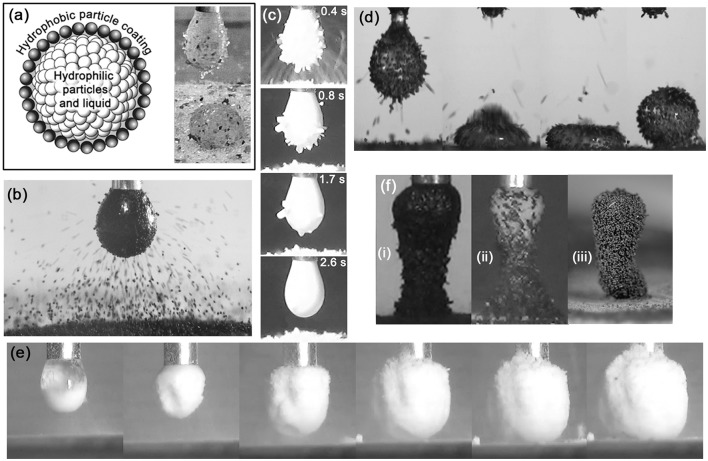
**(A)** Schematic of a complex liquid marble, and CLM formed from silica and sphalerite particles (Jarrett et al., 2016b). **(B)** Aggregate being formed with 80 μm-sized PS/PPy (chloride ion doped) particles (Thomas et al., 2018). **(C)** Transfer to and internalization of PDEA-PS particles by a pH 5.6 droplet, with driving potential of 2.5 kV (Ireland et al., 2018). **(D)** Formation of a liquid marble with 90–125 μm diameter cinnamon particles. **(E)** Formation of large “fluffy” structure with edible CaCO_3_ particles. **(F)** Tower structures formed with (i) Coal particles (ii) Coal and silica (iii) 80 μm-sized PS/PPy (chloride ion doped) particles.

LMs are conventionally formed by rolling a drop of liquid on a bed of hydrophobic particles, which adhere to the air-liquid interface. Clearly, the aggregates formed in our early experiments with hydrophilic particles cannot be formed by this method, as the liquid will simply soak into the particle bed. It was soon established that less hydrophilic particulate materials such as poly(methyl methacrylate) (PMMA) (Jarrett et al., 2014), coal, and sphalerite ((Zn,Fe)S) (Jarrett et al., 2016a) could be transferred to a drop by the electrostatic process, and that these remained at the air-water interface instead of being wetted and thus internalized, as the ballotini were in the initial experiment. This raised the prospect of electrostatically-formed “complex liquid marbles” (CLMs), consisting of a liquid-drop core with internalized hydrophilic particles, surrounded and stabilized by a shell of hydrophobic particles (Figure 2A). These were first produced with internalized silica particles and a shell of sphalerite particles (Jarrett et al., 2016b). The hypothesized core-shell structure was confirmed by capturing, resin-casting and sectioning individual marbles (Jarrett et al., 2016a). One can readily conceive of a variety of applications for a structure of this type, e.g., delivery and controlled release of pharmaceutical powders and water-efficient washing/collection of powder contaminants. The controlled manufacture of these sorts of structured aggregates has been a focussing goal of our subsequent research.

## The electrostatic transfer process

During the electrostatic formation process, the capillary (and thus drop) were earthed via an electrometer, which recorded the flow of negative charge carried to the drop by the particles and thence to earth. Here, and in later experiments, it was not always clear whether the forces transferring particles from the bed to the drop were primarily due to their simply being imparted a net charge by the biased substrate via the bed, or primarily dielectrophoretic forces (where polarized particles are attracted to the region of stronger electric field where the field lines converge on the drop, as shown in Figure 1) (Jones, 2003). In the ballotini experiment already described, a substantial quantity of charge (over 200 μC·kg^−1^) was measured flowing from the drop to earth during particle transfer, confirming an initial net charge on the particles, despite the relatively low conductivity of soda-lime glass (~10^−10^ S·m^−1^) (Grayer, 1996). Our group is currently working on numerical models of the electric field geometry and forces on the particles, a task complicated by the deformation of the drop in the field, which may itself be complicated by the presence of particles at the air-water interface.

The influence of particle surface conductivity has been studied directly using polystyrene (PS) particles carrying a shell of polypyrrole (PPy), which can be doped to conduct electricity (Figure 2B). Since the core component (PS) remains the same for doped and de-doped particles, so do the density, dielectric constant, and other bulk properties. Comparison of doped and de-doped PS/PPy particles has shown that at larger values of the driving potential, the conductive particles are easier to extract from the bed than the non-conductive particles. It is not clear what mechanism is involved here—since conductivity determines the rate of charge transfer, it seems likely that it involves rearrangement of charge during ejection of the particle from the bed (Thomas et al., 2018). Surface conductivity is also thought to affect the rate at which particles discharge after being transferred to the drop, and via this, the rate at which particles jump to the drop once transfer has initiated.

In the meanwhile, however, some qualitative observations and hypotheses can be made about the mechanics of the system. It is apparent that the ability of particles to jump to the drop from the particle bed is not merely a matter of the electrostatic lifting force overcoming their weight, and that interparticle cohesion and friction are also extremely important. Some of these cohesive effects, e.g., van der Waals attraction and mechanical entanglement/structural locking, are present in the absence of an external electric field. Others, such as dielectrophoretic cohesion (Moncada-Hernandez et al., 2014), come into play due to the electric field between the biased substrate and the drop. We have had little success transferring very fine powders (less than ~5 μm), presumably due to cohesion. When these fine powders do jump to the drop, they tend to do so as grains composed of many particles, as observed for the PDEA-PS particles discussed later in this paper.

One curious attribute of the original ballotini experiments was the avalanche phenomenon (Liyanaarachchi et al., 2013). As the particle bed gradually approached the drop, one may have expected the particles to be gradually stripped from the top of the bed. Instead, apart from one or two individual precursors, the particles all leapt from the bed to the drop in an explosive cascade lasting less than half a second once some critical drop-bed separation was reached. The converging electric field geometry shown in Figure 1 exerts a radially-inward force on the bed in the horizontal plane, in addition to the larger vertical force. The bed is essentially being squeezed horizontally inwards at the same time as it is being lifted vertically. This is in some ways analogous to the way a vertical load on an arch “locks” the structure together, except that the shape of an arch distributes a uniaxial vertical force horizontally, whereas in the present system the field itself has a horizontal component. In both cases, there is a limit to the vertical load the structure can bear without failing, and the avalanche occurs at this point. For a powder, in the simplest case, this point is given by the Mohr-Coulomb criterion (Labuz and Zang, 2012), according to which the shear and normal stress at yield have a positive linear relationship (i.e., for a greater compressive stress, a greater shear stress is required for yield). A quantitative mechanical model of the electrostatic stress in the bed, assuming a Mohr-Coulomb yield criterion, confirms this mechanism (Ireland et al., 2015).

## Particle-drop interactions

It is worth noting that materials such as PMMA, sphalerite and coal are not “hydrophobic” in the sense of having a water contact angle greater than 90°. Thus, while they are not dispersible in the liquid as silica is, and would be expected to stay at the interface, they should nonetheless reside more than half immersed in the liquid phase. One might therefore not expect to form genuine stable LMs with these particulate materials, since they do not properly encapsulate the liquid. This was observed with PMMA, which did not form stable marbles, but not with coal and sphalerite, which did. PMMA particles have reported contact angle values of ~65°–85° (Briggs et al., 1990; Ma et al., 2007), compared to ~75°–90° for sphalerite particles (Subrahmanyam et al., 1996) and ~70°–90° for coal, depending on the carbon content (Keller, 1987). The difference appears to be that the PMMA particles were spherical, whereas the sphalerite and coal were more angular in shape, with sharp edges and points at which the air-water contact line could be pinned. These and other irregularly-shaped particles have demonstrated repeatedly that the ability to penetrate the air-water interface is dependent on much more than merely wettability. This applies particularly to this electrostatic formation process, which tends to be more “gentle” than direct mechanical drop-bed contact.

Experiments performed with PS particles provided further insight into these phenomena (Ireland et al., 2016). While these had a hydrophilic surface chemistry, their surface roughness and aggregation behavior tended to produce a metastable Cassie-Baxter wetting regime (Murakami and Bismarck, 2010), which allowed them to form stable liquid marbles. This can be seen almost as a microscale equivalent of the macroscopic morphological irregularity of the sphalerite and coal particles. Perhaps most interesting were the differences between these liquid marbles and those formed by direct mechanical contact such as rolling. It had previously been generally accepted that the maximum ratio of particle to drop diameter required to form stable liquid marbles by direct contact methods was ~1:25 (Eshtiaghi and Hapgood, 2012). Using the electrostatic formation method, stable PS liquid marbles could be formed with particle to drop diameter ratios of up to ~1:12. In addition, in some cases, a very thick, stable particle shell formed, quite unlike the thinner layer observed on rolled marbles. It is supposed that these unique properties of electrostatically-formed LMs are due to the relatively quiescent, shear-free character of the process, compared to direct-contact formation, which is more likely to detach large particles and disrupt thicker shell structures as they form.

## Stimulus-responsive particles

As already mentioned, the controlled manufacture of structured aggregates has been a focussing goal of our group's research effort. A potentially useful tool in controlling the process is the use of stimulus-responsive materials. A variety of studies have been carried out on the direct-contact formation of liquid marbles using materials whose wettability can be triggered by variation in pH, temperature, light, and other stimuli (Fujii et al., 2016b). Our group has begun to extend these studies to electrostatically-formed aggregates, using PS particles carrying a pH-responsive poly[2-(diethylamino)ethyl methacrylate] (PDEA) steric stabilizer on their surfaces (Ireland et al., 2018). PDEA displays hydrophilic behavior below pH ~7 and hydrophobic behavior above pH ~8 (Bütün et al., 2001; Kido et al., 2018). These protonated PDEA-PS particles, which were prepared by drying an acidic dispersion, tend to cohere into grains, in which form they jump from the bed to the drop, instead of being transferred as individual particles. These grains initially adhere to the air-water interface, since the interstices between the particles are full of air. They are then internalized by the drop as water infiltrates the interstices, as shown in Figure 2C. The aggregate formed in this way can thus be seen as a sort of “metastable liquid marble.” Crucially, analysis of the kinetics of the particle transfer and internalization process indicated that it was much more rapid when the drop pH was below 7 than when it was above 8—thus achieving the desired result. Our group is currently extending this work on stimulus-responsive materials by studying electrostatic aggregation using particles coated with a temperature-sensitive polymer.

## Edible particles, exotic structures

Even more remarkable phenomena occurred during attempts to form liquid marbles and other aggregates using edible particles. Food and pharmaceutical applications of simple and complex liquid marbles were prominent in our group's thinking since the beginning of this work, but many of them are complex and difficult to characterize physically and chemically. On the other hand, this complexity can result in rich behavior during electrostatic aggregation. For instance, experiments were performed with mildly hydrophobic cinnamon and hydrophilic edible calcium carbonate. The cinnamon consisted largely of elongated needle-like structures. These tended to align with the electric field and form end-to-end chains, often stretching unbroken from the drop to the cinnamon particle bed—an effect often observed in elongated dielectric particles, such as grass seeds (Jones, 2003). Since the electric field lines near the drop surface were normal to the air-water interface, the cinnamon particles tended to stick straight out once attached, like a pincushion. This had the further, unexpected result of producing extraordinarily stable liquid marbles for a very low interface coverage, as shown in Figure 2D. The outward-pointing needle-like particles maintained a substantial separation between the drop and the particle bed, even under extreme deformation, despite the air-water interface being clearly visible between the particles! Individual calcium carbonate particles were 20 μm spheres with rough surfaces, but these tended to break into more complex structures under moderate shear stress. Figure 2E shows the result of one experiment, in which an extraordinary “fluffy” structure, much larger than the original droplet, was formed. Literature values for the dielectric constant of calcium carbonate are 8.0–9.0 (CRC Handbook of Chemistry and Physics, 2005)—several times those for the other materials mentioned in this paper, which are all around 1.0–3.0. This relatively high dielectric constant may have contributed to the observed behavior by encouraging dielectrophoretic chaining (Jones, 2003). In any case, it is difficult to see how the observed structures formed with cinnamon and calcium carbonate particles could have been produced by rolling of a drop on a particle bed.

We have already seen that irregularly-shaped particles can form stable liquid marbles even when their surface chemistry is hydrophilic. Another striking result of irregular shape is the formation of self-supporting tower-like structures during the particle transfer process (Jarrett et al., 2016b). Figure 2F shows several of these. In the case of coal and sphalerite, the particles are unable to penetrate the air-water interface and accumulate on the outside of the drop. This accumulation is most pronounced on the underside of the drop, where the electric field intensity is greatest and the particle transfer path the shortest, and a stalactite-like protrusion starts to form. The protrusion further concentrates the electric field and shortens the particle transfer path, meaning that subsequent particles preferentially jump to the end of the protrusion, which continues to lengthen until it meets the particle bed and forms a pillar of the sort shown in Figure 2F(i). In many cases this structure continues to stand even when detached from the capillary. Figure 2F(ii) shows an even more complex structure which combines features of particle towers and complex liquid marbles as described above. It was produced by laying a particle bed consisting of a layer of hydrophilic silica on top of a layer of moderately hydrophobic coal. Initially, only silica jumped to the drop, filling it as in the initial ballotini experiments. Coal particles then jumped to the outside of the drop, accumulated on its underside, and formed a pillar as described above. The resulting tower structure resembled a coal wineglass holding a spherical aggregate of silica. These tower structures persisted even after being removed from the capillary and dried. This was not the case with all such particle towers. That shown in Figure 2F(iii) was composed of hydrophilic PS/PPy (chloride ion doped) particles. These were spherical, and thus without the interlocking structural stability of the coal and sphalerite. Unlike the coal and sphalerite structures, the PS/PPy towers collapsed upon being dried. It is hypothesized that the PS/PPy towers were held together by capillary forces due to the water from the droplet, which had infiltrated the whole structure.

## Conclusions

The experiments we have described are only a small sample of the wide range of particulate materials that can be aggregated in this way, and of physical behaviors and liquid-particle structures that one might create with them. For example, we have only just begun to explore the influence of the mechanical, electrical and chemical properties of the drop liquid on the process. A key lesson from this work has been that the results are *not* limited by the imagination—many of them have been spectacularly unexpected! With such rich possibilities on offer, a critical issue becomes control of the process. We are currently studying how the system geometry, bed composition and structure and stimulus-responsive materials can be used to produce a precise and readily replicable aggregate structure with a desired set of properties. These studies aim to refine a demonstrably powerful and flexible aggregate-manufacturing process into a practical and useful tool for a variety of industries.

## Author contributions

All authors contributed to writing the article. SF synthesized the particles unless otherwise noted. CT and BL performed experiments not published elsewhere.

### Conflict of interest statement

The authors declare that the research was conducted in the absence of any commercial or financial relationships that could be construed as a potential conflict of interest.
